# RETRACTION: Impact of Surgical Site Infection After Open and Laparoscopic Surgery Among Paediatric Appendicitis Patients: A Meta‐Analysis

**DOI:** 10.1111/iwj.70360

**Published:** 2025-03-17

## Abstract

**RETRACTION**: LiuJ. and WangQ. , “Impact of Surgical Site Infection after Open and Laparoscopic Surgery Among Paediatric Appendicitis Patients: A Meta‐Analysis,” International Wound Journal 21, no. 4 (2024): e14524, 10.1111/iwj.14524.38084057 PMC10961035

The above article, published online on 12 December 2023, in Wiley Online Library (http://onlinelibrary.wiley.com/), has been retracted by agreement between the journal Editor in Chief, Professor Keith Harding; and John Wiley & Sons Ltd. Following an investigation by the publisher, all parties have concluded that this article was accepted solely on the basis of a compromised peer review process. The editors have therefore decided to retract the article. The authors did not respond to our notice regarding the retraction.

**RETRACTION**: J. Liu and Q. Wang , “Impact of Surgical Site Infection after Open and Laparoscopic Surgery Among Paediatric Appendicitis Patients: A Meta‐Analysis,” International Wound Journal 21, no. 4 (2024): e14524, 10.1111/iwj.14524.38084057 PMC10961035

The above article, published online on 12 December 2023, in Wiley Online Library (http://onlinelibrary.wiley.com/), has been retracted by agreement between the journal Editor in Chief, Professor Keith Harding; and John Wiley & Sons Ltd. Following an investigation by the publisher, all parties have concluded that this article was accepted solely on the basis of a compromised peer review process. The editors have therefore decided to retract the article. The authors did not respond to our notice regarding the retraction.

